# Supplementary material for: Trends in Exclusive, Dual and Polytobacco Use among U.S. Adults, 2014–2019: Results from Two Nationally Representative Surveys
Source: Int J Environ Res Public Health. 2021 Dec 11;18(24):13092. doi: 10.3390/ijerph182413092 (PMC8701855; doi:10.3390/ijerph182413092)
Supplement: Supplementary file 1 [file ijerph-18-13092-s001.zip › ijerph-1490111-supplementary.pdf]

Figure S1. Population prevalence of exclusive, dual and polytobacco use among U.S. adults from 2014/2015 to 2018/2019

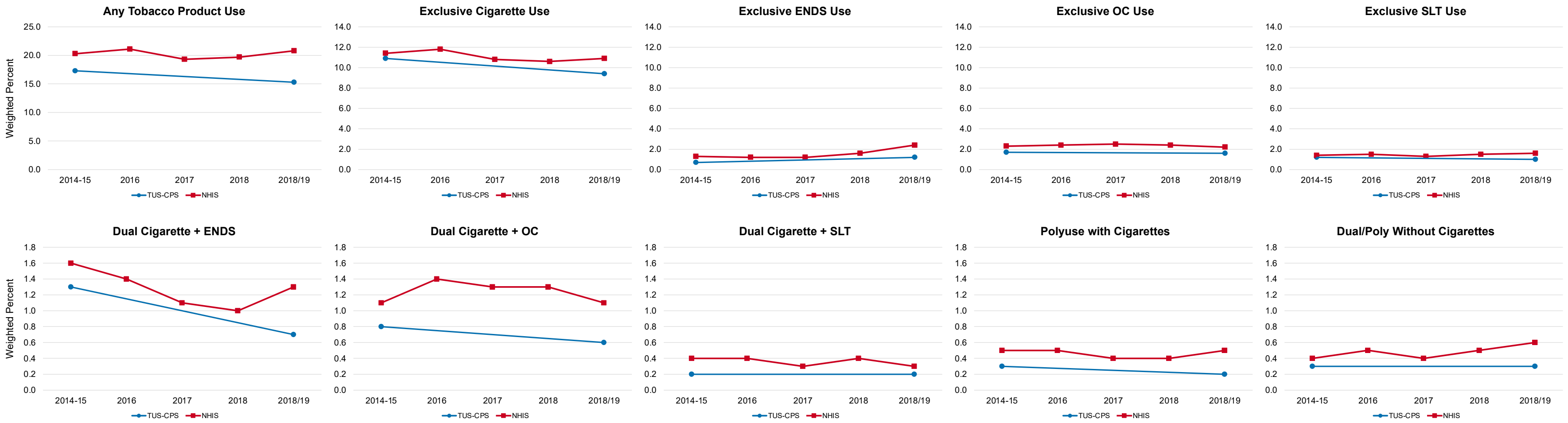

ENDS: Electronic nicotine delivery systems; OC: Other combustible tobacco products; SLT: Smokeless tobacco products

Tobacco Use Supplement to the Current Population Survey (TUS-CPS) sample size: 157,535 (2014-15); 130,826 (2018-19)

National Health Interview Survey (NHIS) sample size: 33,672 (2015); 33,028 (2016); 26,742 (2017); 25,417 (2018); 31,997 (2019)

Table S1. Sample characteristics for the Tobacco Use Supplement to the Current Population Survey (TUS-CPS) and the National Health Interview Survey (NHIS), 2014/15-2018/19

	TUS-CPS				NHIS									
	2014/15		2018/19		2015		2016		2017		2018		2019	
	%	n	%	n	%	n	%	n	%	n	%	n	%	n
Age														
18-24 years	13.0	10483	12.2	7415	12.3	2890	12.1	3024	11.9	2328	11.7	1857	11.7	2165
25-34 years	17.7	25359	18.0	20478	17.6	5783	17.7	5075	17.7	4181	17.9	3905	17.9	4893
35-54 years	34.2	53464	32.6	40788	34.3	10850	33.7	10103	33.4	8137	33.0	7731	32.4	9752
55+ years	35.2	65761	37.2	60077	35.8	14149	36.4	14826	37.0	12096	37.4	11924	38.0	15187
Sex														
Female	51.9	85712	51.8	70257	51.8	18601	51.8	18037	51.8	14646	51.7	13867	51.7	17261
Male	48.1	69355	48.2	58501	48.2	15071	48.2	14991	48.2	12096	48.3	11550	48.3	14733
Race/ethnicity														
NH White	64.8	113288	62.9	93773	65.5	21080	65.0	23374	64.6	18841	63.8	17569	63.4	21918
NH Black	11.7	15451	11.9	12239	12.0	4574	12.1	3626	12.2	2936	12.2	2948	11.8	3483
Another race/ethnicity	7.9	16370	16.6	14052	6.9	2427	7.1	2222	7.3	1721	7.7	1721	8.5	2450
Hispanic	15.6	9958	8.6	8694	15.6	5591	15.8	3806	16.0	3244	16.3	3179	16.4	4091

TUS-CPS: Tobacco Use Supplement to the Current Population Survey; NHIS: National Health Interview Survey

Table S2. Population prevalence of exclusive, dual and polytobacco use among US adults from 2015 to 2019 in NHIS

	Year										
	2015		2016		2017		2018		2019		
**Tobacco product use**	%	95% CI	%	95% CI	%	95% CI	%	95% CI	%	95% CI	P-value
Any tobacco product	20.33	19.66-21.00	21.07	20.30-21.84	19.31	18.59-20.02	19.73	19.00-20.47	20.80	20.10-21.40	0.367
Exclusive cigarette	11.36	10.86-11.86	11.83	11.25-12.41	10.82	10.29-11.36	10.63	10.11-11.14	10.85	10.38-11.33	0.143
Exclusive ENDS	1.26	1.09-1.43	1.19	1.02-1.36	1.19	1.00-1.38	1.64	1.44-1.85	2.35	2.14-2.58	**<0.001**
Exclusive OC	2.33	2.07-2.58	2.44	2.19-2.69	2.45	2.21-2.69	2.39	2.14-2.65	2.23	2.02-2.46	0.552
Exclusive SLT	1.41	1.19-1.62	1.52	1.34-1.70	1.33	1.15-1.50	1.52	1.31-1.72	1.57	1.40-1.77	0.246
Dual cigarettes + ENDS	1.62	1.42-1.81	1.36	1.19-1.53	1.10	0.95-1.26	0.98	0.84-1.13	1.25	1.10-1.43	**0.005**
Dual cigarettes + OC	1.09	0.94-1.24	1.38	1.20-1.56	1.33	1.12-1.54	1.27	1.09-1.46	1.10	0.97-1.24	0.950
Dual cigarettes + SLT	0.37	0.26-0.47	0.37	0.28-0.46	0.31	0.23-0.38	0.40	0.31-0.49	0.30	0.23-0.38	0.287
Polyuse with cigarettes	0.53	0.41-0.64	0.50	0.39-0.60	0.39	0.30-0.49	0.44	0.34-0.54	0.48	0.38-0.60	0.554
Dual/Polyuse w/o cigarettes	0.37	0.27-0.47	0.47	0.35-0.60	0.38	0.29-0.47	0.46	0.36-0.56	0.63	0.51-0.77	**0.002**

NHIS: National Health Interview Survey; ENDS: Electronic nicotine delivery systems; OC: Other combustible tobacco products; SLT: Smokeless tobacco products

Bolded text indicates statistical significance (p<0.05)

Table S3. Population prevalence of any and exclusive tobacco use among US adults from 2015 to 2019 by age group in NHIS

	Year										
	2015		2016		2017		2018		2019		
**Tobacco product use**	%	95% CI	%	95% CI	%	95% CI	%	95% CI	%	95% CI	P-value
Any tobacco product											
18-24	21.47	19.40-23.69	20.35	18.28-22.59	18.28	16.32-20.42	17.11	14.96-19.50	18.13	16.25-20.17	**0.024**
25-34	24.40	22.78-26.11	24.27	22.58-26.04	24.03	22.45-25.68	24.23	22.61-25.94	25.85	24.28-27.48	0.218
35-54	23.37	22.20-24.58	24.96	23.69-26.28	21.38	20.21-22.59	22.61	21.33-23.95	24.54	23.43-25.68	0.162
55+	15.07	14.25-15.93	16.14	15.30-17.03	15.51	14.69-16.36	15.86	15.04-16.71	15.94	15.19-16.72	0.135
Exclusive cigarette											
18-24	8.43	7.10-9.99	8.75	7.43-10.28	6.47	5.28-7.91	4.70	3.61-6.16	4.51	3.66-5.56	**<0.001**
25-34	13.15	12.07-14.30	12.10	10.91-13.41	11.39	10.34-12.52	11.31	10.12-12.62	11.91	10.78-13.13	0.133
35-54	13.55	12.63-14.53	14.21	13.24-15.25	12.82	11.93-13.77	12.87	11.96-13.85	13.09	12.22-14.01	0.488
55+	9.44	8.78-10.14	10.52	9.77-11.33	10.16	9.48-10.88	10.17	9.49-10.88	10.41	9.79-11.06	**0.041**
Exclusive ENDS											
18-24	2.39	1.73-3.30	1.71	1.18-2.48	2.74	1.88-3.98	5.07	3.96-6.46	5.46	4.47-6.66	**<0.001**
25-34	1.56	1.19-2.05	1.58	1.15-2.17	1.92	1.45-2.55	2.52	2.01-3.17	3.57	2.97-4.28	**<0.001**
35-54	1.35	1.10-1.66	1.57	1.26-1.95	0.97	0.76-1.23	1.14	0.89-1.44	2.37	2.05-2.75	**<0.001**
55+	0.64	0.48-0.85	0.48	0.36-0.64	0.54	0.40-0.71	0.59	0.46-0.77	0.80	0.65-0.98	0.201
Exclusive OC											
18-24	4.07	3.20-5.17	2.75	2.06-3.67	2.59	1.93-3.47	2.62	1.87-3.66	1.85	1.28-2.65	**<0.001**
25-34	3.02	2.41-3.78	3.16	2.56-3.89	3.55	2.91-4.33	3.07	2.47-3.80	2.62	2.00-3.41	0.418
35-54	2.06	1.66-2.55	2.64	2.24-3.11	2.40	2.01-2.86	2.31	1.87-2.84	2.76	2.37-3.21	**0.023**
55+	1.64	1.36-1.98	1.81	1.50-2.17	1.91	1.64-2.24	2.07	1.78-2.40	1.70	1.47-1.98	0.766
Exclusive SLT											
18-24	1.49	0.98-2.25	1.84	1.36-2.50	1.44	0.93-2.23	0.77	0.48-1.24	1.14	0.69-1.90	0.423
25-34	1.54	1.15-2.06	1.28	1.00-2.65	1.49	1.13-1.97	1.52	1.13-2.05	1.73	1.30-2.31	0.568
35-54	1.71	1.38-2.13	2.08	1.75-2.47	1.64	1.36-1.98	2.16	1.79-2.61	2.10	1.79-2.45	0.131
55+	1.02	0.76-1.38	1.01	0.84-1.21	0.93	0.74-1.18	1.17	0.95-1.45	1.18	0.97-1.44	0.413

NHIS: National Health Interview Survey; ENDS: Electronic nicotine delivery systems; OC: Other combustible tobacco products; SLT: Smokeless tobacco products

Bolded text indicates statistical significance (p<0.05)

Table S4. Population prevalence of dual and polytobacco use among US adults from 2015 to 2019 by age group in NHIS

	Year										
	2015		2016		2017		2018		2019		
**Tobacco product use**	%	95% CI	%	95% CI	%	95% CI	%	95% CI	%	95% CI	P-value
Dual cigarettes + ENDS											
18-24	1.53	1.10-2.14	1.71	1.15-2.52	0.98	0.63-1.53	0.92	0.57-1.48	1.35	0.87-2.08	0.636
25-34	1.48	1.10-1.98	1.45	1.09-1.93	1.98	1.53-2.55	1.60	1.19-2.14	2.19	1.74-2.74	**0.035**
35-54	2.26	1.92-2.65	1.79	1.50-2.13	1.16	0.91-1.47	1.22	0.98-1.51	1.60	1.34-1.92	**0.006**
55+	1.11	0.88-1.39	0.80	0.65-1.00	0.68	0.52-0.89	0.51	0.38-0.67	0.48	0.38-0.62	**<0.001**
Dual cigarettes + OC											
18-24	0.85	0.52-1.40	1.26	0.84-1.90	1.52	1.02-2.26	1.05	0.59-1.86	1.12	0.72-1.71	0.419
25-34	1.61	1.22-2.13	2.07	1.57-2.72	1.87	1.32-2.62	1.73	1.29-2.32	1.16	0.83-1.61	0.131
35-54	1.24	0.99-1.56	1.50	1.20-1.86	1.40	1.12-1.75	1.48	1.17-1.86	1.24	1.01-1.51	0.992
55+	0.78	0.59-1.02	0.97	0.79-1.21	0.95	0.76-1.18	0.94	0.74-1.18	0.94	0.78-1.14	0.242
Dual cigarettes + SLT											
18-24	0.84	0.42-1.67	0.33	0.18-0.59	0.49	0.26-0.91	0.24	0.11-0.49	0.19	0.07-0.53	**0.037**
25-34	0.57	0.34-0.96	0.87	0.57-1.33	0.56	0.36-0.88	0.75	0.52-1.09	0.48	0.31-0.74	0.626
35-54	0.31	0.21-0.48	0.40	0.27-0.59	0.32	0.22-0.47	0.50	0.34-0.73	0.42	0.29-0.60	0.307
55+	0.15	0.08-0.29	0.12	0.07-0.20	0.11	0.06-0.20	0.19	0.12-0.30	0.14	0.09-0.23	0.871
Polyuse with cigarettes											
18-24	1.04	0.64-1.71	0.91	0.50-1.63	0.94	0.61-1.46	0.86	0.49-1.53	0.85	0.47-1.54	0.595
25-34	1.03	0.68-1.54	0.75	0.50-1.11	0.68	0.42-1.08	0.76	0.52-1.11	1.03	0.72-1.45	1.000
35-54	0.45	0.32-0.64	0.47	0.33-0.66	0.32	0.22-0.47	0.48	0.34-0.68	0.48	0.35-0.66	0.794
55+	0.18	0.10-0.30	0.27	0.18-0.40	0.14	0.08-0.27	0.12	0.07-0.20	0.10	0.06-0.20	0.231
Dual/Polyuse w/o cigarettes											
18-24	0.81	0.48-1.36	1.09	0.66-1.80	1.10	0.67-1.80	0.85	0.49-1.46	1.66	1.11-2.46	**0.033**
25-34	0.45	0.27-0.77	0.99	0.62-1.58	0.59	0.40-0.87	0.97	0.67-1.39	1.18	0.86-1.60	**0.001**
35-54	0.44	0.28-0.69	0.32	0.21-0.47	0.35	0.24-0.52	0.46	0.31-0.67	0.48	0.33-0.68	0.775
55+	0.12	0.06-0.24	0.16	0.09-0.28	0.08	0.04-0.14	0.10	0.05-0.18	0.18	0.10-0.30	0.347

NHIS: National Health Interview Survey; ENDS: Electronic nicotine delivery systems; OC: Other combustible tobacco products; SLT: Smokeless tobacco products

Bolded text indicates statistical significance ($p<0.05$)

Table S5. Population prevalence of any and exclusive tobacco use among US adults from 2015 to 2019 by sex in NHIS

	**Year**										
	2015		2016		2017		2018		2019		
**Tobacco product use**	%	95% CI	%	95% CI	%	95% CI	%	95% CI	%	95% CI	P-value
Any tobacco product											
Female	15.38	14.65-16.13	15.42	14.62-16.25	14.19	13.38-15.04	14.06	13.28-14.87	15.69	14.95-16.46	0.560
Male	25.64	24.56-26.75	27.14	26.06-28.25	24.81	23.79-25.86	25.83	24.75-26.93	26.15	25.24-27.09	0.481
Exclusive cigarette											
Female	11.20	10.58-11.85	11.43	10.74-12.15	10.53	9.80-11.27	10.33	9.68-11.01	10.76	10.16-11.39	0.325
Male	11.53	10.84-12.26	12.27	11.44-14.15	11.14	10.46-11.86	10.95	10.26-11.68	10.94	10.29-11.62	0.233
Exclusive ENDS											
Female	0.89	0.72-1.11	0.96	0.78-1.17	1.11	0.88-1.41	1.16	0.96-1.40	1.93	1.66-2.23	**<0.001**
Male	1.66	1.40-1.96	1.44	1.19-1.75	1.27	1.02-1.58	2.16	1.81-2.57	2.81	2.49-3.16	**<0.001**
Exclusive OC											
Female	0.84	0.65-1.09	0.61	0.46-0.79	0.61	0.45-0.83	0.71	0.55-0.92	0.69	0.53-0.88	0.253
Male	3.92	3.49-4.40	4.41	3.94-4.93	4.42	3.99-4.90	4.19	3.72-4.72	3.87	3.47-4.32	0.880
Exclusive SLT											
Female	0.13	0.09-0.21	0.20	0.12-0.36	0.18	0.11-0.28	0.13	0.07-0.23	0.17	0.10-0.27	0.538
Male	2.77	2.37-3.24	2.93	2.59-3.31	2.57	2.24-2.95	3.00	2.62-3.43	3.07	2.73-3.47	0.298

NHIS: National Health Interview Survey; ENDS: Electronic nicotine delivery systems; OC: Other combustible tobacco products; SLT: Smokeless tobacco products

Bolded text indicates statistical significance (p<0.05)

Table S6. Population prevalence of dual and polytobacco use among US adults from 2015 to 2019 by sex in NHIS

	Year										
	2015		2016		2017		2018		2019		
**Tobacco product use**	%	95% CI	%	95% CI	%	95% CI	%	95% CI	%	95% CI	P-value
Dual cigarettes + ENDS											
Female	1.51	1.29-1.78	1.31	1.12-1.53	0.99	0.82-1.19	0.82	0.68-1.00	1.23	1.04-1.46	0.085
Male	1.73	1.46-2.04	1.42	1.17-1.71	1.23	1.02-1.50	1.16	0.94-1.43	1.27	1.05-1.54	**0.018**
Dual cigarettes + OC											
Female	0.50	0.37-0.66	0.54	0.42-0.70	0.48	0.36-0.65	0.55	0.40-0.76	0.53	0.42-0.67	0.739
Male	1.72	1.47-2.02	2.28	1.96-2.65	2.24	1.86-2.70	2.04	1.75-2.39	1.70	1.46-1.98	0.901
Dual cigarettes + SLT											
Female	0.02	0.01-0.06	0.06	0.03-0.11	0.02	0.01-0.07	0.06	0.03-0.12	0.05	0.02-0.10	0.354
Male	0.73	0.55-0.98	0.71	0.55-0.92	0.60	0.50-0.80	0.76	0.60-0.97	0.57	0.44-0.73	0.201
Polyuse with cigarettes											
Female	0.18	0.10-0.32	0.17	0.11-0.25	0.18	0.11-0.30	0.18	0.10-0.32	0.16	0.10-0.26	0.731
Male	0.90	0.71-1.14	0.85	0.67-1.09	0.62	0.47-0.81	0.72	0.57-0.92	0.82	0.64-1.06	0.618
Dual/Polyuse w/o cigarettes											
Female	0.09	0.05-0.17	0.14	0.06-0.34	0.09	0.04-0.18	0.12	0.06-0.22	0.19	0.11-0.31	0.075
Male	0.68	0.51-0.91	0.83	0.64-1.08	0.70	0.54-0.90	0.83	0.65-1.06	1.10	0.88-1.36	**0.008**

NHIS: National Health Interview Survey; ENDS: Electronic nicotine delivery systems; OC: Other combustible tobacco products; SLT: Smokeless tobacco products

Bolded text indicates statistical significance (p<0.05)

Table S7. Population prevalence of any and exclusive tobacco use among US adults from 2015 to 2019 by race/ethnicity in NHIS

	Year										
	2015		2016		2017		2018		2019		
**Tobacco product use**	%	95% CI	%	95% CI	%	95% CI	%	95% CI	%	95% CI	P-value
Any tobacco product											
Hispanic	13.03	11.88-14.28	13.65	12.06-15.41	12.74	11.45-14.16	13.76	12.21-15.47	13.14	11.91-14.46	0.909
NH White	22.81	21.91-23.74	23.26	22.44-24.10	21.54	20.70-22.40	21.95	21.11-22.81	23.29	22.52-24.07	0.433
NH Black	21.20	19.46-23.05	21.44	19.35-23.70	20.30	18.51-22.21	19.23	17.34-21.26	20.70	19.02-22.49	0.691
Another race/ethnicity	11.78	10.12-13.66	16.97	14.80-19.39	12.22	10.41-14.30	14.86	12.77-17.23	16.31	14.39-18.44	**0.001**
Exclusive cigarette											
Hispanic	8.50	7.57-9.52	8.55	7.33-9.96	8.18	7.17-9.33	7.88	6.79-9.12	6.91	6.03-7.90	**0.021**
NH White	12.27	11.61-12.95	12.55	11.90-13.23	11.69	11.04-12.37	11.44	10.84-12.07	11.86	11.29-12.45	0.367
NH Black	12.77	11.51-14.14	13.33	11.82-14.99	11.46	10.02-13.07	11.62	10.2-13.21	12.34	10.98-13.83	0.662
Another race/ethnicity	6.84	5.67-8.24	10.04	8.55-11.74	7.88	6.37-9.70	8.15	6.53-10.13	8.71	7.25-10.43	0.072
Exclusive ENDS											
Hispanic	0.95	0.63-1.44	0.59	0.37-0.94	1.04	0.54-1.96	1.29	0.83-1.99	1.72	1.32-2.24	**0.012**
NH White	1.50	1.29-1.74	1.37	1.17-1.60	1.33	1.13-1.57	1.93	1.67-2.23	2.63	2.37-2.92	**<0.001**
NH Black	0.57	0.36-0.90	0.67	0.29-1.56	0.84	0.47-1.49	0.76	0.45-1.27	1.63	1.12-2.35	**0.002**
Another race/ethnicity	0.90	0.49-1.65	1.77	1.01-3.07	0.82	0.41-1.64	1.42	0.88-2.26	2.41	1.67-3.45	**0.004**
Exclusive OC											
Hispanic	1.72	1.30-2.28	2.04	1.44-2.87	1.26	0.86-1.82	2.23	1.63-3.06	2.16	1.55-3.01	0.319
NH White	2.39	2.08-2.74	2.58	2.30-2.90	2.70	2.40-3.04	2.45	2.18-2.77	2.20	1.96-2.47	0.358
NH Black	3.36	2.61-4.32	3.03	2.32-3.95	3.45	2.64-4.50	3.07	2.37-3.98	3.23	2.57-4.04	0.811
Another race/ethnicity	1.34	0.85-2.11	1.03	0.63-1.67	1.13	0.68-1.87	1.14	0.74-1.75	1.23	0.81-1.85	0.785
Exclusive SLT											
Hispanic	0.26	0.12-0.53	0.18	0.08-0.41	0.51	0.29-0.89	0.25	0.10-0.64	0.19	0.10-0.35	0.526
NH White	1.97	1.67-2.31	2.08	1.84-2.34	1.75	1.53-2.01	2.06	1.81-2.35	2.20	1.95-2.48	0.260
NH Black	0.46	0.31-0.70	0.44	0.23-0.84	0.50	0.27-0.91	0.55	0.28-1.11	0.39	0.24-0.63	0.589
Another race/ethnicity	0.29	0.13-0.67	1.20	0.62-2.32	0.75	0.38-1.46	1.20	0.65-2.19	1.12	0.65-1.93	**0.013**

NHIS: National Health Interview Survey; ENDS: Electronic nicotine delivery systems; OC: Other combustible tobacco products; SLT: Smokeless tobacco products

Bolded text indicates statistical significance ($p<0.05$)

Table S8. Population prevalence of dual and polytobacco use among US adults from 2015 to 2019 by race/ethnicity in NHIS

	Year										
	2015		2016		2017		2018		2019		
**Tobacco product use**	%	95% CI	%	95% CI	%	95% CI	%	95% CI	%	95% CI	P-value
Dual cigarettes + ENDS											
Hispanic	0.69	0.49-0.98	0.59	0.30-1.16	0.61	0.37-1.00	0.87	0.56-1.36	0.59	0.37-0.93	0.570
NH White	1.93	1.67-2.23	1.70	1.49-1.93	1.37	1.19-1.59	1.13	0.96-1.34	1.49	1.27-1.73	**0.015**
NH Black	1.28	0.87-1.86	0.55	0.35-0.85	0.69	0.41-1.14	0.56	0.36-0.89	1.04	0.67-1.63	0.502
Another race/ethnicity	1.34	0.81-2.22	1.40	0.86-2.25	0.51	0.24-1.08	0.69	0.34-1.39	1.07	0.64-1.79	0.540
Dual cigarettes + OC											
Hispanic	0.49	0.33-0.72	1.21	0.80-1.83	0.89	0.52-1.54	0.73	0.41-1.30	0.90	0.62-1.30	**0.036**
NH White	1.08	0.91-1.27	1.30	1.12-1.52	1.28	1.09-1.51	1.28	1.08-1.52	1.14	0.98-1.33	0.610
NH Black	2.37	1.85-3.03	2.35	1.69-3.26	2.45	1.72-3.48	1.95	1.44-2.64	1.26	0.88-1.79	**0.003**
Another race/ethnicity	0.39	0.20-0.73	0.82	0.41-1.63	0.82	0.45-1.50	1.26	0.67-2.36	0.93	0.60-1.43	**0.024**
Dual cigarettes + SLT											
Hispanic	0.04	0.01-0.13	0.06	0.02-0.17	0.06	0.02-0.16	0.13	0.03-0.67	0.13	0.05-0.35	0.225
NH White	0.53	0.39-0.71	0.54	0.42-0.69	0.45	0.35-0.58	0.56	0.45-0.70	0.42	0.33-0.54	0.285
NH Black	0.09	0.03-0.27	0.07	0.02-0.22	0.03	0.01-0.23	0.05	0.01-0.19	0.04	0.01-0.15	0.389
Another race/ethnicity	0.05	0.02-0.15	0.10	0.03-0.36	0.04	0.01-0.18	0.16	0.07-0.36	0.04	0.01-0.15	0.738
Polyuse with cigarettes											
Hispanic	0.24	0.12-0.51	0.20	0.08-0.49	0.14	0.06-0.33	0.15	0.05-0.41	0.27	0.13-0.53	0.856
NH White	0.66	0.51-0.84	0.60	0.47-0.76	0.47	0.36-0.62	0.54	0.42-0.70	0.60	0.46-0.78	0.600
NH Black	0.16	0.08-0.31	0.50	0.27-0.91	0.41	0.20-0.82	0.24	0.11-0.55	0.20	0.10-0.42	0.654
Another race/ethnicity	0.58	0.17-1.92	0.26	0.14-0.48	0.23	0.09-0.55	0.54	0.24-1.21	0.40	0.20-0.83	0.651
Dual/Polyuse w/o cigarettes											
Hispanic	0.14	0.05-0.35	0.23	0.09-0.60	0.06	0.02-0.20	0.24	0.10-0.59	0.27	0.14-0.55	0.242
NH White	0.50	0.38-0.67	0.54	0.40-0.73	0.48	0.38-0.62	0.54	0.43-0.69	0.75	0.60-0.94	**0.027**
NH Black	0.15	0.06-0.37	0.51	0.27-0.97	0.47	0.19-1.16	0.43	0.18-0.99	0.58	0.30-1.11	**0.035**
Another race/ethnicity	0.05	0.01-0.18	0.36	0.12-1.01	0.05	0.01-0.21	0.32	0.12-0.86	0.40	0.18-0.89	**0.033**

NHIS: National Health Interview Survey; ENDS: Electronic nicotine delivery systems; OC: Other combustible tobacco products; SLT: Smokeless tobacco products

Bolded text indicates statistical significance ($p<0.05$)
